# The Pivotal Role of Airway Smooth Muscle in Asthma Pathophysiology

**DOI:** 10.1155/2011/742710

**Published:** 2011-12-11

**Authors:** Annaïg Ozier, Benoit Allard, Imane Bara, Pierre-Olivier Girodet, Thomas Trian, Roger Marthan, Patrick Berger

**Affiliations:** ^1^Univ. Bordeaux, Centre de Recherche Cardio-Thoracique de Bordeaux, F-33000 Bordeaux, France; ^2^INSERM U1045, F-33000 Bordeaux, France; ^3^CHU de Bordeaux, Service D'Exploration Fonctionnelle Respiratoire, CIC-P 0005, F-33000 Bordeaux, France

## Abstract

Asthma is characterized by the association of airway hyperresponsiveness (AHR), inflammation, and remodelling. The aim of the present article is to review the pivotal role of airway smooth muscle (ASM) in the pathophysiology of asthma. ASM is the main effector of AHR. The mechanisms of AHR in asthma may involve a larger release of contractile mediators and/or a lower release of relaxant mediators, an improved ASM cell excitation/contraction coupling, and/or an alteration in the contraction/load coupling. Beyond its contractile function, ASM is also involved in bronchial inflammation and remodelling. Whereas ASM is a target of the inflammatory process, it can also display proinflammatory and immunomodulatory functions, through its synthetic properties and the expression of a wide range of cell surface molecules. ASM remodelling represents a key feature of asthmatic bronchial remodelling. ASM also plays a role in promoting complementary airway structural alterations, in particular by its synthetic function.

## 1. Introduction 

The pathophysiology of asthma is characterized by the association of airway hyperresponsiveness (AHR), inflammation, and remodelling [1–3]. AHR is defined by an increased airway narrowing to a wide range of stimuli and is responsible for recurrent episodes of wheezing and breathlessness. Airway smooth muscle (ASM) is considered as the main cell type involved in AHR [4, 5]. Bronchial inflammation in asthma involves the recruitment of various inflammatory cells including eosinophils, mast cells and T lymphocytes [1]. However, the microlocalization of these cell types is different within the asthmatic ASM layer [6], suggesting complex interactions between inflammatory cells and ASM cells. Bronchial remodelling is described as an increased thickening of the bronchial wall due to various structural alterations including epithelial changes [7], subepithelial membrane thickening, enhanced extracellular matrix (ECM) deposition [8], mucous gland and goblet cell hypertrophy and hyperplasia [9], neoangiogenesis [10], and increase in ASM mass [11, 12]. This latter appears to be a key feature of bronchial remodelling since increased ASM mass is associated with a decrease in lung function in asthma [13–15]. The aim of the present article is thus to review the pivotal role of ASM in the pathophysiology of asthma.

## 2. Role of ASM in Airway Hyperresponsiveness

AHR is usually described as either nonspecific or specific AHR (Table 1). Nonspecific AHR is a common feature of asthma, although it is also found in some patients suffering from chronic obstructive pulmonary disease or allergic rhinitis [16]. Stimuli inducing nonspecific AHR can be direct or indirect (Table 1). Direct mediators stimulate ASM cell membrane receptors. For instance, methacholine activates muscarinic M3 receptor and induces ASM contraction [17]. Conversely, indirect mediators first stimulate one or more intermediary cells, leading to the release of contractile agonists, which, in turns, induce ASM contraction [18]. All of these indirect challenges are associated with the release of mast cell mediators, such as prostaglandins, PGD2 and PGF2*α*, or histamine, which, respectively, activate ASM cell membrane receptors TP, FP, and H1 [19]. For instance, acute and intense exercise induces airway dehydration, increasing osmolarity of the airway surface liquid [20]. Cell volume shrinkage and restoration release both mast cell mediators and acetylcholine from nerves [20]. On the other hand, specific AHR is limited to allergic asthmatics. In such case, the stimulus is an allergen against which the patient is specifically sensitized. The mechanism of ASM contraction also involves the release of mast cell mediators. Specific AHR measurement is restricted to clinical trials or detection of occupational diseases. Indeed, allergen-specific stimulation can enhance nonspecific AHR [21] and induce a late phase response [19].

The mechanisms of AHR in asthma may involve (i) a larger release of contractile mediators, (ii) a lower release of relaxant mediators, (iii) an improved ASM cell excitation/contraction coupling, and/or (iv) an alteration in the contraction/load coupling.

### 2.1. Larger Release of Contractile Mediators

The contraction of ASM can be induced by a variety of extracellular messengers, which act through interaction with specific receptors at the site of the plasma membrane of ASM (Table 2). These contractile agonists, either inflammatory mediators or neurotransmitters, are released at high concentrations in asthmatic bronchi, especially regarding mast cell mediators following indirect challenges [19]. For instance, mast cells release tryptase, which increases ASM cells cytosolic calcium concentration [30], allowing them to contract after activation of the protease activated receptor (PAR) type 2, expressed on their membrane [31]. Mast cells, but also eosinophils, produce contractile prostaglandins such as PGF2*α*, PGD2, and thromboxane TXA2. The concentration of these prostaglandins, and also that of histamine, is increased in asthma [32]. Similarly, inflammatory cells also produce both cysteinyl leukotrienes (LTC4, LTD4, LTE4), which are also very potent direct contractile agonists of ASM [33], and LTB4, which acts indirectly through the release of TXA2 [34]. Such cysteinyl leukotrienes are also increased in asthma [35], as well as endothelin [36], another direct contractile agonist of ASM [37], produced by epithelial cells or endothelial cells [38]. The release of contractile neurotransmitters, acetylcholine or tachykinins, is also known to be higher in asthma [39, 40]. It is noteworthy that the loss of epithelial barrier integrity commonly associated with asthma may increase exposure of ASM to inhaled contractile agonists [41].

### 2.2. Lower Release of Relaxant Mediators

AHR may also be related to a lower release of relaxant agonists either neurotransmitters or inflammatory mediators (Table 2). Physiologically, the adrenergic innervation is sparse in the ASM [51]. Moreover, a decreased number of *β*2-adrenergic receptor sometimes associated with its desensitization has been suggested in asthma following chronic medication with short-acting *β*2-agonists [52]. A lower release of adrenaline has also been demonstrated during asthma attacks [53]. The second important relaxing neurotransmitter is the vasoactive intestinal peptide (VIP), which is colocalized with acetylcholine [54]. However, the expression of VIP in the asthmatic ASM is not decreased but surprisingly increased [55]. By contrast, the production of PGE2, which is known to relax ASM, is significantly lower in asthmatic than in nonasthmatic ASM cells [56]. Other cellular sources of PGE2 and other ASM relaxant, such as NO, may also be implicated.

### 2.3. Improved ASM Cell Excitation/Contraction Coupling

Cytokines, such as IL-5 or IL-13, that are both increased in asthma, do not contract ASM *per se*. In fact, IL-5 induces *in vitro* AHR to acetylcholine in isolated rabbit ASM tissue and IL-13 reduces responsiveness to adrenaline in human ASM cells [57, 58]. Moreover, mast cell-derived tryptase induces AHR to histamine *in vitro* using human bronchi from actively sensitized [59] or nonsensitized patients [60] possibly *via* its enzymatic activity. However, such AHR occurs after an initial calcium response and, thus, in the absence of any spontaneous contraction. Indeed, AHR may also depend on excitation/contraction coupling, which associates two subsequent steps within the ASM cell, that is, (i) the calcium response induced by extracellular messengers and (ii) the calcium sensitivity of the contractile apparatus. 

**Figure 1:**
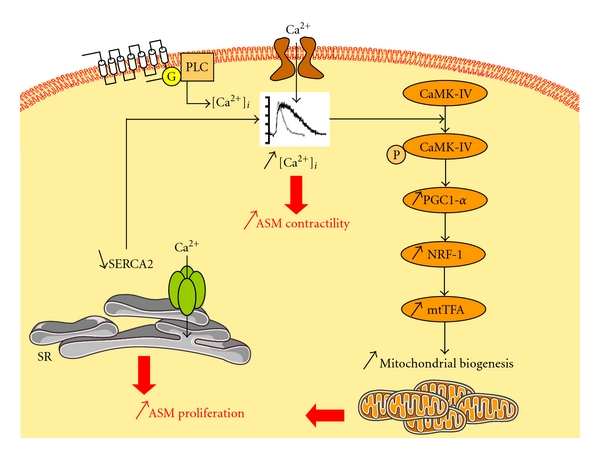
Impaired ASM cell calcium homeostasis leading to enhanced proliferation in asthma. In severe asthmatic ASM cells, an altered calcium homeostasis related to an increased influx leads to phosphorylation and activation of CaMK-IV, that, in turns, successively activates PGC-1*α*, NRF-1, and mtTFA. This transduction pathway results in an increase of mitochondrial biogenesis leading to enhanced ASM proliferation [61]. In nonsevere asthmatic ASM cells, an altered expression and function of SERCA2 may account for the altered calcium homeostasis, which leads to enhanced ASM proliferation [62]. Whatever the mechanism, such altered calcium homeostasis enhances cell contractility. ASM: airway smooth muscle; CaMK-IV: calcium/calmodulin-dependent protein kinase IV; G: G protein; mtTFA: mitochondrial transcription factor A; NRF: nuclear respiratory factor; PGC: peroxisome proliferator-activated receptor *γ* coactivator; PLC: phospholipase C; SERCA: sarcoendoplasmic calcium pomp; SR: sarcoplasmic reticulum.

On the one hand, following extracellular stimulation, ASM cytosolic calcium homeostasis is dependant on various components. For instance, the spontaneous return to baseline of the cytosolic calcium concentration is dramatically delayed in asthmatic ASM cells [61]. Such a delay can be related with an abnormal calcium entry [61] and/or with a downregulated expression and function of type 2 of the sarcoendoplasmic calcium pump, that is, SERCA2 [62] (Figure 1). SERCA2 protein expression could be experimentally decreased by IL-13 or TNF-*α* (TNFSF2) [63]. Alternatively, the proinflammatory cytokine, IL-1*β*, can increase CD38 (ADP-ribosyl cyclase) expression. Then, CD38 increases cyclic ADP-ribose (cADPR) production, which, in turns, activates ryanodin receptor (RyR), leading to an increase in cytosolic calcium concentration [64–66]. In addition, calcium compartmentalization could be another determinant of airway responsiveness. In asthmatic ASM, there could be a greater proportion of intracellular calcium within the deep cytosolic space as compared to the superficial subplasmalemmal space [67], leading to AHR. However, such hypothesis needs to be confirmed.

On the other hand, the calcium sensitivity of the contractile apparatus can be increased, leading to a higher contractile response after a similar ASM intracellular calcium rise [68]. Two different mechanisms have been described according to the dependency or the independency from the myosin light chain kinase/myosin light chain phosphatase (MLCK/MLCP) ratio (Table 3). Regarding MLCK/MLCP-dependent AHR, the level of MLCK is increased in asthmatic ASM [69, 70]. The transcription factor CCAAT/enhancer binding protein-*α* (C/EBP-*α*) expression is decreased within the asthmatic ASM [71]. Since the promoter that regulates the expression of MLCK contains several C/EBP-*α* binding sites, such a deficit in C/EBP-*α* has been proposed to account for the increased expression of MLCK [72]. Nevertheless, such increased expression of MLCK in asthmatic ASM needs to be confirmed since controversial findings have also been reported [73, 74]. Regarding MLCK/MLCP-independent AHR, calcium-independent PKC*ε* inhibits calponin, an actin thin filament-associated protein [75] that decreases calcium sensitivity [76]. It also activates mitogen-activated protein kinase (MAPK), which inhibits caldesmon, another actin thin filament-associated protein [75] that also decreases calcium sensitivity [76]. Alternatively, RhoA/Rho-kinase signalling, which is increased in experimental asthma, inactivates MLCP leading to MLCK/MLCP-dependent AHR [77], and also inhibits calponin leading to MLCK/MLCP-independent AHR. Such RhoA signalling can be reproduced experimentally, using either the proinflammatory cytokine TNF-*α* (TNFSF2), which activates RhoA [78], or IL-13, which increases its expression in murine ASM [79].

Finally, the alteration in the dynamic properties of ASM in asthma is still a matter of current debate. The maximal velocity of shortening in ASM from sensitized animals is significantly greater than those in nonsensitized [80] and may be a consequence of an increase in the activity of MLCK [81] but is still controversial (see above). With respect to force generation, although isometric force generated *in vitro* is directly related to ASM mass in human bronchial ring preparations [82], force generation from asthmatic ASM was not found consistently increased [83]. 

### 2.4. Alteration in Contraction/Load Coupling

Like any muscle, ASM may overcome loads that normally moderate ASM shortening [98]. Two main categories of mechanical loads thus apply to ASM: a preload, which is directly related to lung elastic recoil and lung volume, and an afterload, which is determined by the shear modulus of the parenchyma and the coupling of the lung to the airways [98]. Some of these loads reside at the airway cellular/tissular level. Any alteration in such loads against which the ASM contracts and/or in the application of these loads to ASM will result in an alteration in shortening and, hence, airway narrowing. 

In asthma, several mechanisms may account for an alteration in the contraction/load coupling [83, 98] such as an increase in lung elastic recoil pressure and a lung expansion that increase the external load that opposes ASM shortening. Indeed, loss of lung elastic recoil related to hyperinflation has been demonstrated in acute asthma [99] and chronic persistent asthma even without emphysema [100]. Moreover, a coupling between lung parenchyma and airways occurs through the attachments of elastic fibers to the airway wall [98]. In healthy individuals, periodic deeper inspirations (DIs) dilate the airways because of an excessive relative airway hysteresis as compared to parenchymal one [101]. In contrast, loss of the beneficial effect of DI is sometimes considered as a hallmark of asthma [102, 103]. Why DI induces bronchoconstriction in some asthmatic patients remains a matter of debate. Leading hypotheses put forward include neurohormonal mechanisms such as increase in cholinergic tone [104] or mechanical mechanisms such as calcium-dependent myogenic response of ASM [105] or loss of interdependence between lung parenchyma and airways [106]. Such uncoupling of the forces of interdependence may also be the result of airway inflammation and remodelling, especially thickening and/or oedema of the adventitia [107–109]. Moreover, stiffness of the airway wall may reduce the ability of tidal breathing and DI to stretch ASM, leading to latch bridges between actin/myosin and to a vicious positive feedback [110, 111]. Finally, altered organization and degradation of ECM proteins might also be less effective to moderate ASM shortening [83, 112, 113].

On the other hand, thickening of the airway wall and the enhanced amount of ECM also increase airway stiffness and decrease airway compliance [114] that might enable the airways to resist to dynamic compression. Moreover, deposition of connective tissue also acts as mechanical impedance to contraction [113]. Nevertheless, these theoretical advantages of airway remodelling are largely overwhelmed by their negative effects [83]. 

## 3. Role of ASM in Bronchial Inflammation

While early studies regarding ASM function in asthma focussed on its contractile properties, a growing body of evidence now consistently demonstrates that ASM is no longer a sole target of the inflammatory process. Indeed, ASM cell also displays proinflammatory and immunomodulatory functions [115, 116], through its synthetic function and its expression of a wide range of cell surface molecules, integrins, costimulatory molecules, and Toll-like receptors (Table 4). As a consequence, ASM can play an active role, through an autocrine and/or a paracrine manner, in the asthmatic inflammatory process. The molecular and cellular mechanisms modulating ASM cell/inflammatory cell function will be reviewed below, with special attention to those implicating mast cells and T lymphocytes, which are known to infiltrate the asthmatic ASM layer [6].

### 3.1. ASM/Mast Cell Interaction

There is evidence that the asthmatic ASM layer is infiltrated by an increased number of mast cells [6, 55, 130, 140–147]. Some mast cells are already present in nonasthmatic ASM including smokers [148–152]. However, this mast-cell microlocalization within the asthmatic ASM, termed mast cell myositis [153], appears to be a specific feature of asthma, being absent in patients suffering from eosinophilic bronchitis and healthy subjects [140, 145]. Interestingly, the mast cell myositis is observed in various asthma phenotypes, including eosinophilic and noneosinophilic asthma [154], and also atopic and non atopic asthma, even if the number of mast cells is significantly higher in the ASM of atopic asthmatics [142]. Moreover, mast cell myositis is unchanged whether asthma is treated or not [145, 146], or regardless of asthma severity [143, 145–147].

**Figure 2:**
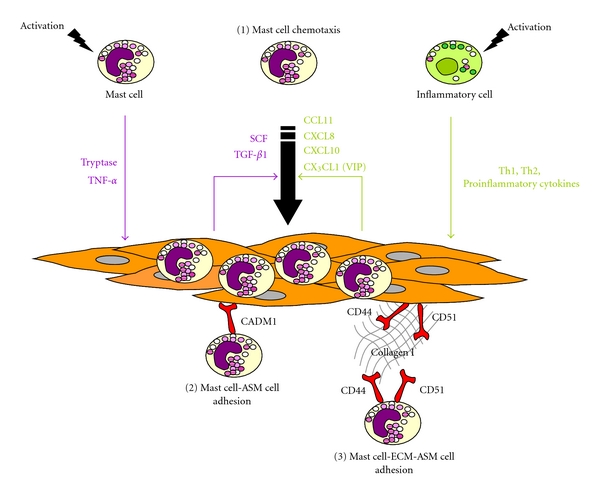
Mechanisms underlying mast cell myositis in asthma. In asthma, the mechanisms leading to an infiltration of airway smooth muscle (ASM) layer by mast cells, termed mast cell myositis, involve (1) mast cell chemotaxis towards the ASM bundle, (2) direct mast cell-ASM cell adhesion, and (3) mast cell-extracellular matrix- (ECM-) ASM cell adherence. Upon mast cell activation, mast cells release mediators which activate ASM cells, such as tryptase and TNF-*α* (TNFSF2). As a consequence, ASM cells produce and secrete chemotactic factors for mast cells, leading to an autoactivation loop. Under stimulation by Th1, Th2, and/or proinflammatory cytokines, produced by various inflammatory cells, ASM cells also secrete a wide range of mast cell chemotactic factors.

The mechanism of such a myositis has been firstly related to the production of mast cell chemotactic factors by the ASM itself, through an autoactivation loop [144] (Figure 2). Indeed, upon activation, mast cells release tryptase and proinflammatory cytokines, such as TNF-*α* (TNFSF2), which stimulate the production of TGF-*β*_1_ and, to a lesser extent, SCF by ASM cells, which in turns, induce mast cell chemotaxis [144]. Moreover, ASM can promote mast cell chemotaxis through the secretion of a wide array of chemoattractants, upon stimulation by Th1 [130], Th2 [130, 155] or proinflammatory cytokines [55, 144]. For instance, ASM also produces functionally active CXCL10 [130], CXCL8 [155], CCL11 [155], and CX_3_CL1 [55], even if, for CX_3_CL1, the additional presence of VIP is necessary [55]. Taken together, these findings support the view that mast cell migration depends on various mediators secreted by ASM and is closely related to ASM inflammatory microenvironment. 

 Once present within the ASM bundle, mast cells can adhere to ASM. This adhesion has been initially ascribed to a cell-cell interaction involving an Ig superfamily member, that is, cell adhesion molecule 1 (CADM1), previously known as tumor suppressor in lung cancer 1 (TLSC-1 ) [156, 157] (Figure 2). However, blocking CADM1 leads to only a partial reduction in the adhesion of mast cells to ASM, suggesting additional mechanisms are present [156]. Indeed, mast cell-ASM adherence also involves cell-ECM-cell interaction through type I collagen, CD44, and CD51 [2] (Figure 2). This adhesion is improved under inflammatory conditions or using asthmatic ASM cells [2]. These *in vitro* findings are in agreement with ultrastructural analysis of asthmatic ASM using electron microscopy, demonstrating the absence of direct cell-cell contact between ASM and mast cells [6].

The majority of mast cells infiltrating the asthmatic ASM layer are typically of the MC_TC_ phenotype, containing both tryptase and chymase [140, 158, 159]. Interestingly, these mast cells infiltrate ASM in both large and small airways, and exhibit marked features of chronic ongoing activation [142, 143]. Such findings were also confirmed by ultrastructural analysis of ASM using electron microscopy [6]. However, little is known about the mechanisms by which mast cell activation may occur within the ASM layer [160]. Mast cell degranulation may result from IgE-dependent activation, especially in atopic patients [142]. However, IgE-independent mechanisms have also been evoked, following mast cell-ASM interaction through the complement C3a or SCF [42, 157, 161, 162], for instance, or following bacterial or viral infection through Toll-like receptors [160]. 

Taking into account the following characteristics: (i) microlocalization of mast cells within the ASM layer, (ii) mast cell adherence to the ASM, and (iii) mast cell activation within the ASM, it is tempting to consider that a close functional relationship may exist between these two cell types. 

On the one hand, mast cells alter functional and phenotypic properties of ASM cells. Indeed, mast cell-derived mediators contribute to AHR and ASM remodelling [12]. For instance, the major mast cell product, tryptase, induces both ASM calcium response [30] and AHR to histamine *in vitro* [60] or *in vivo *[163]. Tryptase also increases ASM cell-TGF-*β*1 secretion, which, in turns, promotes ASM cells differentiation towards a contractile phenotype, characterized by an increase expression of *α*-actin and enhanced contractility [42]. Interestingly, the number of mast cells within the ASM layer is positively correlated with the degree of AHR [140, 145], and with the intensity of *α*-smooth muscle actin [42]. 

Mast cell myositis may also promote ASM remodelling. However, although several mast cell products such as tryptase are known to individually stimulate both DNA synthesis and ASM proliferation [164], coculturing ASM and mast cells does not increase proliferation [161]. Similarly, ASM survival is not enhanced by mast cell interaction [161]. Both mast and ASM cell-derived CCL19 mediate ASM migration through ASM CCR7 activation [128]. By contrast, CCL11/CCR3-mediated ASM cell migration was inhibited by mast cells [165]. No correlation was found between the number of mast cells in the ASM and ASM mass, supporting the modest role of mast cells in ASM remodelling [142]. 

On the other hand, ASM cells alter functional and phenotypic properties of mast cells. Indeed, ASM cells can promote mast cell survival, hence providing a favorable microenvironnement for mast cells [157]. They also enhance their proliferation, through a mechanism involving a coo-perative interaction between ASM membrane-bound SCF, soluble IL-6, and mast cell-expressed CADM1 [157]. ASM cells-derived ECM proteins may also promote mast cells differentiation towards a fibroblastoid phenotype, charac-terized by the expression of fibroblast markers and fibroblast-like morphology. This feature seems to be specific of mast cells within the ASM layer, since fibroblast markers are not expressed in submucosal mast cells [147].

### 3.2. ASM/T Cell Interaction

Up to now, only few studies have documented T cell infiltration within the ASM layer in asthma. CD4^+^ T cell microlocalization within ASM layer was first reported in an elegant experimental rat asthma model [166]. This finding was further confirmed in human asthmatics [6, 15] and is related to asthma severity [15]. Moreover, this feature appears to be specific of asthma, since T cells were not found in the ASM layer of control subjects, but within and beneath the epithelium [15]. As compared to mast cell ASM infiltration, there is a relative paucity of T cells within the ASM layer [6, 140]. However, ASM cells are able to produce appropriate chemotactic factors for T cells, such as CCL5 [125]. In this respect, a possible role for mast cell chymase has been suggested since this protease is known to inhibit T cell adhesion to nonasthmatic ASM cells *in vitro* [167]. Nonetheless, close contact were pointed out between ASM cells and T cells in asthma *ex vivo *[6, 15], hence suggesting cell-cell adherence between these two cell types.

Lazaar and coworkers originally demonstrated that activated T cells can adhere *in vitro* to resting ASM cells from nonasthmatic patients and that such an adhesion was enhanced when ASM cells were primed with proinflammatory cytokines such as TNF-*α* (TNFSF2) [118]. These findings were independently further confirmed [117]. This adhesion involves CD44, intercellular cell adhesion molecule 1 (ICAM-1), vascular cell adhesion molecule 1 (VCAM-1) expressed by ASM cells, and CD44, lymphocyte function-associated antigen 1 (LFA-1) and very late antigen 4 (VLA-4) expressed by T cells [118]. More recently, nonasthmatic ASM cells, pulsed to the superantigen staphylococcal enterotoxin A (SEA), have been shown to adhere to T cells by presenting the SEA *via* their MHC class II [168]. Although ASM cells express MHC class II, constitutively as well as under stimulation [117, 120], they are not classically considered as an antigen-presenting cell. Consequently, these findings support an emerging role of ASM cell as an immunomodulatory cell. However, except for VCAM-1, which forms clusters in the asthmatic ASM *ex vivo* suggesting VCAM-1 mediated intercellular signalling, the role of the above molecules in adherence between T cell and ASM cell from asthmatic patients has not yet been considered. Two other ASM cell-surface molecules, CD40 (TNFRSF5) [117, 169–171] and OX40 ligand (TNFSF4) [119, 171, 172], both expressed in asthmatic and nonasthmatic ASM cells, may also play a role in promoting ASM cell-T cell adherence. These costimulatory cell-surface molecules, members of the TNF superfamily, respectively, bind to CD40L (TNFSF5) and OX40 (TNFRSF4) on activated T cells [173, 174]. However, their role in the adherence of T cells to asthmatic ASM remains to be investigated.

Close interaction between T cells and ASM cells may lead to stimulatory cross-talk between these two cell types, but little is known about the functional consequences of such an interaction. On the one hand, T cells may alter functional properties of ASM cells. In this connection, T cells alter ASM contractile phenotype, enhancing ASM contractility to acetylcholine and reducing its relaxation to isoproterenol in isolated rabbit ASM tissue [117]. T cells may also drive ASM remodelling, in particular ASM hyperplasia [15, 118, 166]. Indeed, in an experimental rat asthma model, adoptively transferred CD4^+^ T cells from OVA-sensitized rats increases ASM mass, which is both associated with an increased ASM proliferation and decreased apoptosis *ex vivo *[166]. Such an increased ASM proliferation and decreased apoptosis was confirmed *in vitro *only upon direct CD4^+^ T cells-ASM cells contact, highlighting the need for close cellular interaction between these two cell types [166]. These observations are in agreement with a previous study demonstrating *in vitro* a role for T cell in ASM DNA synthesis and proliferation in ASM cells from nonasthmatic patients [118]. The role of T cells in driving ASM remodelling was later confirmed in human asthmatics [15]. Moreover, the number of T cells infiltrating the asthmatic ASM correlates with ASM mass [15]. Collectively, these findings suggest an emerging role of T cells in both ASM hyperresponsiveness and remodelling. 

On the other hand, ASM cells may also alter functional properties of T cells. Indeed, a direct contact between CD4^+^ T cells and ASM cells also enhances T cell survival, thus possibly contributing to the perpetuation of bronchial inflammation [166]. Moreover, cultured human nonasthmatic ASM cells are able to present superantigens *via* their MHC class II molecules to resting CD4^+^ T cells [168], which leads to CD4^+^ T cells activation, adherence between these cells, and finally release of IL-13, that, in turns, leads to increase in the contractile response to acetylcholine of isolated rabbit ASM tissues [168]. 

## 4. Role of ASM in Bronchial Remodelling

**Figure 3:**
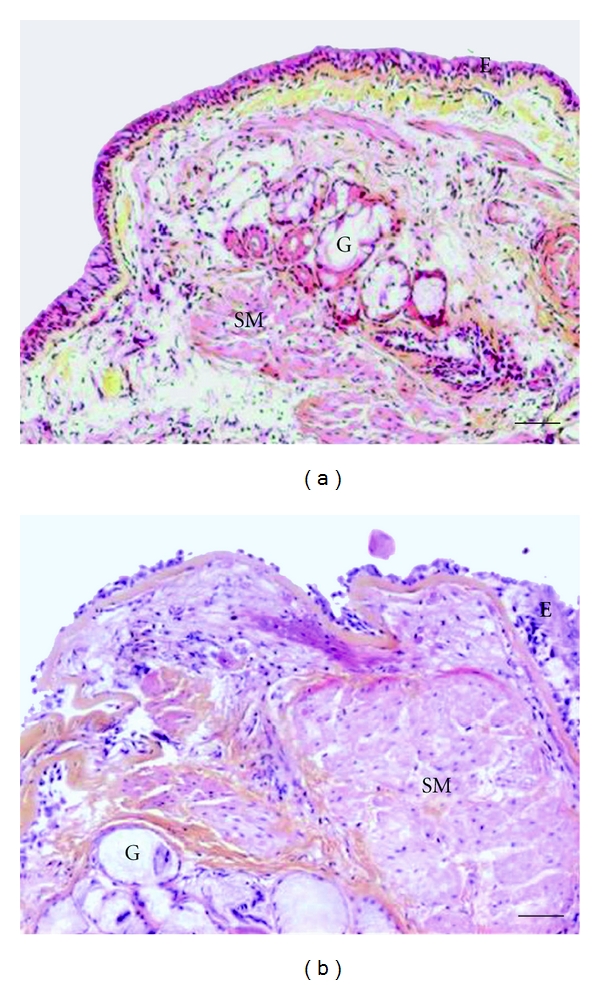
Representative optic microscopic from bronchial sections stained with Haematoxylin, Eosin, and safranin stain were obtained from (a) a control subject or (b) an asthmatic patient (printed from Bara et al. [12], with permission of European Respiratory Journal publisher) E: epithelium; G: mucous gland; SM: smooth muscle. Scale bars represent 50 *μ*m.

ASM remodelling represents a key feature of asthmatic bronchial remodelling [12] (Figure 3). Indeed, many lines of evidence demonstrate an increase in ASM mass in fatal [175] and nonfatal asthma [15, 61, 70, 142]. However, ASM remodelling is still insensitive to current asthma medications [176] that are usually effective in treating acute airway narrowing and bronchial inflammation. Furthermore, ASM cell plays also a role in promoting other bronchial remo-delling structural alterations, in particular by virtue of its synthetic function. For instance, ASM cells can release biologically active TGF-*β* [177], which is involved in various structural alterations such as epithelial changes, subepithelial fibrosis, mucus hypersecretion, goblet cell hyperplasia, and angiogenesis [178]. 

The mechanisms underlying ASM remodelling involve ASM hyperplasia, ASM hypertrophy, and an excessive deposition of proteins of the ECM [12].

### 4.1. ASM Hyperplasia

Three mechanisms may account for ASM hyperplasia, that is, (i) ASM cell proliferation, (ii) reduced ASM cell apoptosis, and more recently (iii) migration of myofibroblasts within the ASM layer.

#### 4.1.1. ASM Cell Proliferation

Compelling evidence now suggests an excessive ASM cell proliferation in asthma both *in vitro* [61, 62, 71, 179, 180] and *in vivo *[15, 181]. However, before these two recent studies [15, 181], the number of proliferative ASM cells *in vivo* was a matter of debate since the number of PCNA or Ki67 positive ASM cells was unchanged in asthmatic bronchial samples [6, 70]. The reason for this discrepancy is apparently related with the fixation process. 

A wide array of mitogenic factors are known to promote ASM cell proliferation *in vitro*, including growth factors, cytokines, chemokines, inflammatory mediators, enzymes, ECM components, reactive oxygen species, and mechanical stress (Table 5). Such mitogenic factors promote ASM cell proliferation by activating pathways involving either receptor tyrosine kinase (RTK) or receptor coupled to heterotrimeric *G* proteins (GPCR) [182]. Upon activation of these receptors, downstream transduction pathways usually involve Ras, phosphatidylinositol 3-kinase (PI3K) or MAPK/ERK kinase (MEK) and extracellular signal-regulated kinase (ERK), which in turns induce cyclin D1 expression [182].

However, asthmatic ASM cells can also proliferate faster *in vitro *than nonasthmatic ASM cells irrespective of extracellular stimuli [179], hence supporting the view that an intrinsic ASM abnormality does exist in asthma. In this respect, ASM tissue-specific decreased levels of C/EBP*α* were found in asthmatics and could explain enhanced proliferation [71], through downregulation of the cell-cycle inhibitor p21. In addition, steroids are less effective in asthmatic ASM cells, since C/EBP*α* expression is impaired [71]. An alternative, or complementary, ASM tissue-specific explanation for the increased asthmatic ASM cell proliferation is an alteration of ASM calcium homeostasis. In severe asthma, an abnormal extracellular calcium entry leads to subsequent activation of peroxisome proliferator-activated receptor *γ* coactivator-1*α* (PGC-1*α*), nuclear respiratory factor-1 (NRF-1), and mitochondrial transcription factor A (mtTFA) [61]. This transduction pathway results in an increased mitochondrial biogenesis leading to enhanced ASM proliferation [61] (Figure 1). Interestingly, such enhanced mitochondrial biogenesis was not observed in other cell types, such as endothelial or epithelial cells, highlighting the smooth muscle specificity of such findings [61]. More recently, ASM calcium homeostasis has also been shown to be impaired in nonsevere asthma, *via* a different mechanism [62]. Such mechanism implicates reduced SERCA2 expression in both native and cultured ASM cells, but origin of such defect in asthmatic ASM remains unknown [183] (Figure 1).

#### 4.1.2. Reduced ASM Apoptosis

Another explanation is to consider that ASM hyperplasia may be due to an imbalance between ASM proliferation and death. However, little is known about processes that may counterbalance ASM proliferation, such as apoptosis. Indeed, by contrast to the substantial evidence supporting ASM proliferation, only few studies have investigated ASM cells survival signals, ASM cell spontaneous apoptosis, or their susceptibility to apoptosis in response to proapoptotic factors. Most of the current knowledge about apoptotic pathways in ASM originates from nonasthmatic ASM cells. 

Cardiotrophin-1 [184], endothelin-1 [185], chemokines, such as CCL3, CCL5, CCL11, and CXCL8 [186], or some ECM components, including fibronectin, laminin, and collagens I and IV [187], inhibit nonasthmatic ASM cell apoptosis. ASM cells express *in vitro* the death receptors TNFR1 (TNFRSF1A), Fas (TNFRSF6), TRAILR1 (TNFRSF10A), TRAILR2 (TNFRSF10B) [188], and stimulation of ASM cells either with TNF-*α* (TNFSF2) [188, 189], soluble Fas ligand (TNFSF6) [188], Fas antibody [189], or TRAIL (TNFSF10) [188] induces cell apoptosis. Similarly, hydrogen peroxide-exposure decreases ASM cells survival and is prevented by overexpression of the small heat shock protein 27 (Hsp27), in part by upregulating glutathione levels [190]. ECM-degrading proteinase, such as neutrophil elastase, may also induce ASM cell apoptosis by triggering detachment from the ECM [191], which results in the withdrawal of survival signals usually provided by some ECM components [187]. Conversely, some ECM components, such as decorin, may also induce ASM cells apoptosis *in vitro* [192]. Some treatments are also able to induce nonasthmatic ASM cell apoptosis *in vitro*. Indeed, activation of peroxisome proliferator-activated receptor *γ* (PPAR*γ*), which is expressed by ASM cells, by its synthetic ligand commonly used in diabetes treatment, may also induce apoptosis [193]. Moreover, simvastatin, an HMG-CoA reductase inhibitor, has recently been shown to trigger ASM cells apoptosis through the intrinsic apoptotic pathway involving p53, enhanced mitochondrial permeability, mitochondrial release of Smac and Omi, and inhibition of mitochondrial fission, which in turns leads to the activation of caspases 9, 7 and 3 [194]. However, the role of these different mediators and signalling pathways in asthmatic ASM cell survival remains unknown.

In asthma, the hypothesis of an imbalance between ASM proliferation and apoptosis is mainly supported by a rat model of T-cell driven remodelling [166]. Furthermore, an increased expression of TRAIL (TNFSF10) has also been shown *ex vivo* in asthmatic ASM following allergen challenge, suggesting that this cytokine may play a role in asthmatic ASM apoptosis [195]. Besides, active caspase 3 expression and double stranded DNA breaking were shown *ex vivo* in ASM, in severe asthma, and in case of fatal asthma, respectively [188]. Conversely, such findings were not reported in intermittent asthma [188] and Benayoun and coworkers failed to demonstrate active caspase 3 expression *ex vivo*, irrespective of asthma severity [196]. *In vitro*, two reports have also drawn negative results, showing no change in spontaneous apoptosis within asthmatic ASM cells [61, 161]. As a consequence, further studies remain to be undertaken to determine whether or not there is a change in asthmatic ASM cell apoptosis.

#### 4.1.3. Migration of ASM Cells and Myofibroblasts

More recently, it has been suggested that migration of ASM precursor cells, either located within the bronchial wall or derived from peripheral blood fibroblast progenitors, towards the ASM bundles may also participate in ASM hyperplasia [197]. Cellular migration is characterized by significant cytoskeletal remodelling with apparition of filopodia and lamellipodia to increase directed movement along a concentration gradient (chemotaxis) and/or nondirected movement (chemokinesis) [197].

Migration of myofibroblasts may participate in ASM hyperplasia, by differentiating to ASM-like cells [197]. Such an hypothesis was based on the microlocalization of myofibroblasts between ASM bundles [6], while an increased number of myofibroblasts has been demonstrated within the lamina reticularis, especially after allergen challenge [198], and within the lamina propria [15]. 

However, the origin of myofibroblasts remains a source of debate and several hypotheses have been put forward. First, myofibroblasts may come from resident fibroblasts that have differentiated [199] or from dedifferenciation of ASM cells themselves, which have migrated out of the ASM bundles towards the lumen [199]. Indeed, a wide range of mediators may promote* in vitro* human nonasthmatic ASM cell migration, such as cytokines, growth factors, chemokines, and some ECM components [197] (Table 6). The major transduction pathways so far identified for ASM cell migration involve PI3K, Rho-kinase, MAPK, p38, and phosphorylation of Hsp27 [197, 200]. Migration is also a strongly calcium-dependent process [62, 128]. Thus, enhanced cell spreading, which might reflect migration, was observed in asthmatic ASM cells lacking SERCA2, while knocking down SERCA2 using small interfering RNA in nonasthmatic ASM cells leads to earlier lamellipodia and enhanced cell spreading [62]. Whereas this first study suggests that asthmatic ASM cells might migrate more than nonasthmatic ASM cells [62], such findings require further investigations, as well as addressing directly ASM cell migration *in vivo*. 

Myofibroblasts can also originate from epithelial cells undergoing phenotypic changes through epithelial-mesenchymal transition process [201–203]. Another hypothesis is that myofibroblasts come from the recruitment of circulating bone-marrow-derived progenitors, termed fibrocytes [204–208]. Fibrocytes may be identified by the coexpression of the stem cell marker CD34 and/or the panhematopoietic marker CD45 and collagen-1 [209]. In this setting, a growing body of evidence supports the concept of fibrocytes trafficking to the lung in asthma, both in mouse asthma models [204, 208] and in humans [204–207]. Indeed, fibrocyte localization has been found within the bronchial wall in asthma [204], in particular, within the ASM bundles irrespective of asthma severity [206], close to the basement membrane [207], in the lamina propria [206], or below the epithelium [204, 208]. Fibrocytes differentiate into myofibroblasts, as evidenced by the concomitant expression of fibrocyte markers and *α*-smooth muscle actin [204, 205]. Mechanisms underlying such fibrocyte chemotaxis to the lung are in part mediated by ASM secreted-PDGF [206], which also promotes fibrocyte differentiation to myofibroblast [205]. Besides, such chemotaxis might likewise involve the chemokine receptors CXCR4, CCR2, CCR3, CCR4, CCR5, CCR7, and the chemokines CCL3, CCL4, CCL5, CCL7, CCL8, CCL11, and CCL13 [210]. Interestingly, circulating fibrocytes number is positively correlated to the slope of the yearly decline in FEV_1_ [205], once again suggesting a role of fibrocytes in ASM remodelling.

### 4.2. ASM Hypertrophy

Unlike ASM cell hyperplasia, evidence for ASM cell hypertrophy in asthma remains a matter of debate [6, 70, 74, 175]. In fact, increased ASM cell size has been reported in tissue specimens from intermittent [70], mild-to-moderate [70], severe [70], fatal [175], and atopic asthma [6]. However, conflicting findings were found in mild-to-moderate asthma [74]. As a consequence, it has been suggested that ASM cell hypertrophy might be a hallmark of severe asthma status, since it can discriminate patients with severe asthma from those with milder disease [70]. Interestingly, ASM cell hypertrophy was associated with an increased amount of MLCK, which is involved in AHR [70]. Nevertheless, whether ASM hypertrophy is a condition sufficient to induce AHR in asthma needs further investigations. 

The underlying mechanisms of such ASM cell hypertrophy remain unknown in asthma. Cardiotrophin-1 [184], serum deprivation [237], TGF-*β* [238], and endothelin-1 [185] can drive ASM cell hypertrophy *in vitro*, and only in nonasthmatic ASM cells. More recently, the role of miRNA-26a, whose expression is increased by mechanical stretch, has been highlighted in ASM cell hypertrophy [239]. However, the role of these factors needs to be clarified in asthma, as well as the putative role of SERCA2, whose expression is known to be also reduced in cardiac hypertrophy [240]. 

Two distinct transduction pathways can lead to ASM cell hypertrophy [241]. Pathways can involve the mammalian target of rapamycin (mTOR), 4E-binding protein (4E-BP), the transcription factor eIF4E [242], and S6 kinase [243] or the inhibition of glycogen synthase kinase- (GSK-) 3*β* [244], for instance, by the serine/threonine kinase Akt [241]. However, involvement of such transduction pathways needs to be further investigated in asthmatic ASM cell hypertrophy.

### 4.3. Altered ECM within the ASM Layer

Alteration in ECM represents another feature of ASM remodelling [12]. ECM is increased in asthma [6], as a result of increased deposition of ECM proteins by airway resident cells, such as epithelial cells, fibroblasts, myofibroblasts, and ASM cells (Table 4). Its composition is different from that of nonasthmatics [139]. Histological examination of asthmatic bronchial samples reveals enhanced deposition of ECM proteins in the bronchial wall, such as collagens I, III, V, fibronectin, tenascin, hyaluronan, versican, laminin, lumican, and biglycan [245, 246], while collagen IV and elastin are decreased [247]. However, conflicting findings have also been reported regarding, the amount of collagen III [248, 249], collagen IV [250] and elastin [251] in the asthmatic bronchial wall. Interestingly, ECM is also increased all around ASM cells regardless of asthma severity [6, 252]. Such increase may be due to decreased matrix metalloproteinases (MMPs), which degrade ECM proteins, and/or increased tissue inhibitors of MMPs (TIMPs). Thus, overexpression of TIMP-1 and TIMP-2 may account for low MMPs activity in asthma [138]. The profile of ECM proteins produced *in vitro* by asthmatic ASM cells is also different from that of nonasthmatic ASM cells, with more perlecan and collagen I, and less laminin-*α*1 and collagen IV [253]. 

Nevertheless, whether such alterations in ASM ECM products contribute to modulate composition of the overall bronchial ECM remains unknown. Conversely, increasing evidence supports the role of ECM in modulating several aspects of ASM function [254]. In fact, fibronectin and collagen I have been reported to promote ASM proliferation from nonasthmatic donors in a dose-dependant fashion (Table 5), whereas laminin inhibits growth [255]. Fibronectin, collagens I, IV, and laminin also provide strong survival signals for ASM cells and such an effect is mediated at least in part through the fibronectin receptor/*α*5*β*1 integrin [187]. Moreover, ASM cell contact with membranes coated with ECM components, such as fibronectin, collagens III and V, has been shown to enhance ASM migration [233] (Table 6). Synthetic properties of ASM cells may also be modulated by ECM components, such as IL-1*β*-dependent ASM secretion of CCL5 and CCL11 [256]. More recently, ECM has been involved in triggering ASM maturation to the contractile phenotype upon serum deprivation [257].

## 5. Conclusion

ASM cell plays a pivotal position in the pathophysiology of asthma as (i) a main effector of AHR, (ii) a proinflammatory and immunomodulatory cell through its synthetic properties and its expression of a wide range of cell surface molecules, and (iii) a leading cell involving in bronchial remodelling. If AHR and bronchial inflammation respond fairly well to conventional therapy such as bronchodilators and anti-inflammatory drugs, ASM remodelling remains insensitive to these treatments [176]. Targeting the various functions of ASM represents therefore a challenge for future asthma treatments [5, 12, 176]. In particular, research needs to focus on ways to prevent and/or reverse ASM remodelling. In this connection, targeting mitochondrial biogenesis and/or developing proapoptotic strategies to induce ASM cell death may represent an attractive area of investigation. In this respect, development of noninvasive tools for quantifying ASM remodelling has received increasing attention in order to evaluate on a long-term basis the efficacy of such treatments [258].

## Figures and Tables

**Table 1:** Stimuli used to assess airway hyperresponsiveness (AHR) *in vivo*.

AHR	Mechanisms	Types	Stimuli
Nonspecific	Direct	Pharmacological	Methacholine [22]
			Histamine [23]

Nonspecific	Indirect	Physical	Exercise [24]
			Cold air, dry air [25]
			Eucapnic hyperventilation [26]
			Hypo osmolar solution [27]

Nonspecific	Indirect	Chemical	Adenosine monophosphate [28]
			Mannitol [27]

Specific	Indirect	Allergen	Pollens, House dust mites [29]

**Table 2:** Contractile and relaxant mediators for ASM.

Types of mediators	Actions	Stimuli
Inflammatory	Contraction	Histamine [23]
		Tryptase [42]
		Prostanoids (PGF2*α*, PGD2, TXA2) [43]
		Cysteinyl leukotrienes (LTC4, LTD4, LTE4) [22]
		Endothelin [44]

Neurotransmitters	Contraction	Acetylcholine [45]
		Neurokinine A [46]
		Substance P [46]
		Calcitonin Gene Related Peptide [47]

Inflammatory	Relaxation	Prostanoids (PGE2, PGI2) [48]

Neurotransmitters	Relaxation	Adrenaline [49]
		Noradrenaline [50]
		Vasoactive Intestinal Peptide [46]

**Table 3:** Excitation/contraction coupling in ASM cells.

MLCK/MLCP balance	Extracellular factors	Intracellular factors	Actions	Consequences	References
Dependent		CaMKII	I: MLCK	R	[84]
	*β*-adrenergic agonist	PKA	I: MLCK	R	[85]
	*β*-adrenergic agonist	PDE4D increase	I: PKA	C	[86]
		PKG	A: MLCP	R	[87]
	TNF-*α* (TNFSF2), IL-13	Rho-kinase	I: MLCP	C	[87]
	TNF-*α* (TNFSF2)	Arachidonic acid	A: Rho kinase	C	[88, 89]
	TNF-*α* (TNFSF2)	Arachidonic acid	I: MLCP	C	[88, 90]
		CPI-17	I: MLCP	C	[87]
		C/EBP-*α* decrease	I: MLCK	C	[72]

Independent		Caldesmon	I: Myosin ATPase activity	R	[91]
		Calponin	I: Myosin ATPase activity	R	[92]
		PKC*ε*	I: Caldesmon	C	[75]
		PKC*ε*	I: Calponin	C	[75]
	Derp1, LPS	MAPK	I: Caldesmon	C	[75, 93, 94]
	TNF-*α* (TNFSF2), IL-13	Rho-kinase	I: Calponin	C	[95]
		CaMKII	I: Calponin	C	[95]
		HSP phosphorylated	I: Contraction	R	[96, 97]

A: active; C: contracting; CaMKII: calcium/calmodulin-dependent protein kinase; CPI-17: 17-kDa PKC-potentiated inhibitory protein of PP1; C/EBP*α*: CCAAT/enhancer binding protein *α*; Derp1: house dust mite allergen; HSP: heat shock protein; I: inhibit; IL-13: interleukin-13; LPS: lipopolysaccharide; MAPK: mitogen-activated protein kinase; MLCK: myosin light chain kinase; MLCP: myosin light chain phosphatase; PDE4D: phosphodiesterase 4D, cAMP-specific; PKA: cAMP-dependent protein kinase A; PKC*ε*: protein kinase C *ε*; PKG: cGMP-dependent protein kinase G; R: relaxing; PP1: protein phosphatase 1; TNF-*α*: tumor necrosis factor *α*.

**Table 4:** Mediators secreted and immunomodulatory proteins expressed by human ASM cells.

Factors	References
*Cell adhesion/costimulatory molecules, receptors*	
CD11a	[117]
CD40 (TNFRSF5), CD40L (TNFSF5), CD44	[118]
CD80, CD86	[117]
OX40L (TNFSF4)	[119]
Adhesion molecules: ICAM-1, VCAM-1	[118]
Major histocompatibility complex (MHC) II	[120]
Toll-like receptors: TLR2, 3, 4	[121]
Chemokine receptors: CCR3, 7	[122]
Chemokine receptors: CXCR1, 3, 4	[122]
Receptors for IL-4, 6, 12, 13, 17, 22 and IFN-*γ*	[116, 123]
*Chemokines, cytokines, and growth factors*	
CCL2, 5, 7, 8, 11, 17, 19	[124–128]
CXCL8, 10	[129, 130]
CX_3_CL1	[55]
IL-2, 5, 6, 11, 12	[57, 131]
Interferon- (IFN-) *γ*	[57]
Connective tissue growth factor (CTGF)	[132]
Granulocyte macrophage-colony stimulating factor (GM-CSF)	[133]
Stem cell factor (SCF)	[134]
Transforming growth factor- (TGF-) *β*1	[135]
Vascular endothelial growth factor (VEGF)	[136]
*ECM proteins*	
Chondroitin sulfate	[137]
Collagens I, III, IV, V	[137]
Decorin, elastin, fibronectin, laminin, perlecan	[137]
Thrombospondin	[137]
Tissue inhibitor of MMPs- (TIMPs-) 1, 2	[138]
*Enzymes*	
Matrix-metalloproteinases- (MMPs-) 9, 12	[139]

**Table 5:** Mitogenic factors for human ASM cells.

Classification	References
*Growth factors/cytokines/chemokines*	
Platelet-derived growth factor (PDGF)	[211]
Fibroblast growth factor (FGF)	[212]
Epidermal growth factor (EGF)	[213]
Tumor necrosis factor- (TNF-) *α* (TNFSF2)	[214]
Transforming growth factor- (TGF-) *β*1 (controversial)	[215, 216]
CCL3, 5, 11	[186]
CXCL8	[186]
*Inflammatory mediators*	
Histamine	[217]
Endothelin-1	[218]
Thromboxane A2	[219]
Sphingosine 1-phosphate	[220]
Cysteinyl leukotrienes (LTC4, LTD4, LTE4)	[221]
*Enzymes*	
Tryptase	[164]
Thrombin	[222]
Elastase	[223]
Matrix metalloproteinases	[224]
*ECM components*	
Fibronectin	[225]
Collagen I	[225]
*Others*	
Reactive oxygen species	[226]
Mechanical stress	[227]
Brain derived neurotrophic factor (BDNF)	[228]

**Table 6:** Promigratory factors for human ASM cells.

Factors	References
*Growth factors/cytokines/chemokines*	
FGF2, PDGF, TGF-*α* (TNFSF2), TGF-*β*	[229, 230]
IL-1*β*, IL-17A, IL-17F, IL-22	[123, 230]
CCL5, 11, 19	[128, 231, 232]
CXCL8	[231]
Leukotriene E4	[233]
*ECM components*	
Collagens I, III, V	[233]
Fibronectin, laminin	[233]
*Enzymes*	
Matrix metalloproteinase- (MMP-)3	[234]
*Others*	
Thrombin	[235]
Urokinase plaminogen activator	[236]
